# Machine learning for accelerating screening in evidence reviews

**DOI:** 10.1002/cesm.12021

**Published:** 2023-07-20

**Authors:** Mary Chappell, Mary Edwards, Deborah Watkins, Christopher Marshall, Sara Graziadio

**Affiliations:** ^1^ York Health Economics Consortium, Enterprise House, Innovation Way University of York York UK

**Keywords:** machine learning, rapid review, record screening, systematic review

## Abstract

Evidence reviews are important for informing decision‐making and primary research, but they can be time‐consuming and costly. With the advent of artificial intelligence, including machine learning, there is an opportunity to accelerate the review process at many stages, with study screening identified as a prime candidate for assistance. Despite the availability of a large number of tools promising to assist with study screening, these are not consistently used in practice and there is skepticism about their application. Single‐arm evaluations suggest the potential for tools to reduce screening burden. However, their integration into practice may need further investigation through evaluations of outcomes such as overall resource use and impact on review findings and recommendations. Because the literature lacks comparative studies, it is not currently possible to determine their relative accuracy. In this commentary, we outline the published research and discuss options for incorporating tools into the review workflow, considering the needs and requirements of different types of review.

## 1 INTRODUCTION

Evidence reviews, including systematic, rapid and scoping reviews, are important for informing decision‐making in clinical practice, where they underpin guidelines and practice decisions. They can also inform primary research, where the synthesis of previous work enables the most efficient design and planning of subsequent studies. Systematic reviews are time‐consuming and costly [1] and keeping up with the increasing rate of research publications can be difficult [2]. Even when conducting rapid reviews, with more pragmatic searches and curtailed methods, a large amount of work is associated and may not be within the scope or budget of many research institutions or companies.

With the advent of artificial intelligence, including machine learning in natural language recognition, used extensively in social media and other contexts, there is an opportunity to apply these methods to evidence reviews [3]. Software tools have been developed for all stages in the review process and there appears to be great potential for review work to be accelerated [4]. One element of the process with many available machine learning applications is study screening, where tools have been developed to identify studies for inclusion and exclusion [5]. However, despite advances in development being made over the last decade, uptake and implementation of automated tools has been slow [6] and skepticism in the systematic review community remains [7]. It has been suggested that “comparability with current practice” is fundamental in limiting tool uptake [7]. If reviewers and decision‐makers are not convinced that tools maintain accuracy compared to current practice, they will not be used. In addition, if tools do not fit with ease into the current workflow, or affect the established processes, then this may inhibit adoption [7].

Here, we discuss the potential use of machine learning for screening in evidence reviews by considering the available tools and how they have been evaluated to date. We then discuss issues relating to their application in different types of evidence review.

## 2 MACHINE LEARNING SCREENING TOOLS FOR SYSTEMATIC REVIEWS

### 2.1 What is available?

The Systematic Review Toolbox [8] holds a comprehensive record of available tools to support the systematic review process, including screening. The function and usability of machine learning tools has been assessed in a number of reviews [6, 9, 10, 11, 12] and some of the tools have been identified as having good usability and acceptability or being rated highly by users. However, in the majority of these reviews [9, 10, 11, 12], the accuracy of machine learning screening tools was not evaluated.

Tools predominately aid screening by using machine learning to rank records according to their likelihood of inclusion. Following a training period (commonly 200–300 records [13, 14]), tools rank the remaining records in order of likelihood of inclusion and users may use automated tool settings, or select a cut‐point in their rankings, to subsequently assign includes and excludes to their review. Another common approach is to continue training and reranking as part of the screening process. In this way, reviewers keep screening within the tool (and the tool keeps reranking) until they are satisfied that they have obtained all, or the majority, of relevant studies.

### 2.2 Are they accurate?

Tool accuracy is, of course, essential for ensuring the validity and acceptability of reviews that leverage these types of applications. Even in a rapid review context, reviewers and review commissioners need some assurance that the tools capture relevant studies and that they subsequently reach appropriate conclusions.

The efficacy of screening tools is considered in terms of diagnostic test accuracy, where sensitivity is the ability of a tool to correctly identify records that should be included in the review, and specificity/workload saved is the ability of a tool to correctly exclude records that do not meet review criteria. As with studies of diagnostic test accuracy, there is a “reference standard,” which is used to establish the true inclusions and exclusions, and this is generally single‐ or dual‐reviewer screening. Studies are retrospective, using past systematic review sets as their “data” to test whether tools can correctly identify the included and excluded studies.

Since the priority for systematic reviews is the correct inclusion of relevant studies (sensitivity), most studies use a “fixed sensitivity” approach, where screening continues until the majority (95%–100%), of included studies have been identified and the specificity, or workload saved, is then calculated.

Published studies have shown workload savings of between 14% and 95% (Table 1). This variation is not fully explained by differences in tool performance since there is also large variation across studies of the same tool (e.g., 30%–99% [22, 23]). Furthermore, estimates of workload saved vary *within* studies when tools are tested on different reviews, in some cases by over 50% [16, 26, 27]. It is clear that the performance of tools can vary widely and authors have noted that accuracy is likely to be lower in reviews with more complex research questions [16] and tools may perform better in larger reviews, where they give bigger workload savings [27].

**Table 1:** Machine learning screening tools.

Tool	Study	Sensitivity	Specificity	Tool use
Abstrackr	Gates 2018 [15]	0.79–0.96^a^	0.19–0.90^a^	Machine ranking/human screening
	Gates 2020 [5]	0.62–1.00^a^	0.85–0.99^a^	Machine ranking/human screening
	Gates 2020 [14]	0.86–1.00^a^	0.30–0.43^a^	Machine ranking/human screening
	Rathbone 2015 [16]	0.98–1.00^a^	0.14–0.64^a^	Machine ranking/human screening
	Giummarra 2020 [17]	0.98	0.83	Continuous machine training and ranking
ASReview Lab	Van de Schoot 2021 [18]	0.95	0.67–0.92^a^	Continuous machine training and ranking
Cochrane RCT classifier	Thomas 2021 [19]	0.99	0.63	Machine ranking/human screening
Concept encoder	Yamada 2020 [20]	1.00	0.87	Continuous machine training and ranking
Dbpedia	Tomassetti 2011 [21]	1.00	0.20	Continuous machine training and ranking
DistillerAI	Burns 2021 [22]	1.00	0.99	Machine ranking/human screening
	Gartlehner 2019 [13]	0.00–0.32^a^	0.97–0.99^a^	Machine ranking/human screening
	Hamel 2020 [23]	1.00	0.30–0.73^a^	Continuous machine training and ranking
Rayyan	Olofsson 2017 [24]	0.86–0.99^a^	0.50	Machine ranking/human screening
Research Screener	Chai 2021 [25]	1.00	0.65–0.95^a^	Continuous machine training and ranking
Robot analyst	Przybyla 2018 [26]	0.95	0.07–0.66^a^	Continuous machine training and ranking
SWIFT‐active screener	Howard 2020 [27]	0.95–1^a^	0.10–0.66^a^	Continuous machine training and ranking

^a^ Range of results for different review test sets.

### 2.3 When and how are they useful?

It appears likely that some machine learning screening tools do have sufficient accuracy to make them potentially useful for evidence reviews. However, the trade‐off between workload savings and lost sensitivity needs to be considered before their use in each new review.

Different types of literature review may require different levels of sensitivity. For pragmatic, or rapid, reviews, a lower level of sensitivity is generally acceptable, where reviewers and commissioners are willing to accept that the review will not include all relevant studies. In other situations, for example in scoping searches, high levels of sensitivity may not be required, as the aim is not to capture all studies but to map the volume and/or type of literature in a particular field. In these cases, machine learning tools may be particularly useful as large workload savings may be achieved. Depending on the desired level of sensitivity, there are several options for incorporating screening tools into a review.

Most available tools allow users to select the number of reviewers they wish to participate in screening (normally 1 or 2). Evaluations in the literature vary, with some using single reviewers to train and/or test tools [5, 14, 15, 16, 20] and some using two reviewers or simulations based on dual reviewer screening [22, 26], but there is no obvious pattern for how accuracy is affected. There is empirical research suggesting that single‐reviewer screening is less accurate than double‐reviewer screening [28, 29], but it is unclear whether the size of this error is negligible in comparison with error already introduced by the use of screening tools. It appears that, even with one reviewer using a tool, high levels of sensitivity are possible (100%) [16, 20]. However, if tool training and ongoing screening is undertaken by two reviewers, it is anticipated that higher levels of sensitivity are likely to be achieved, but with an inevitable decrease in workload saved.

In addition, a team must also decide the most appropriate approach to using the tool, for example, “tool use,” as shown for each study in Table 1.

The machine ranking/human screening approach presented in Table 1 involves training tools on a small set of records (usually 200–300). Once the tools have made their predictions, the records are exported and ranked based on the probability of being included. A cut‐off point is then applied to separate the records into two groups: excludes and possible includes [13, 14, 15].

The possible includes can undergo further review by a human reviewer or can be moved directly into the full‐text screening stage to determine if they meet the eligibility criteria. Where a high cut‐point is applied to exclude the majority of records, this type of approach is likely to be very time‐saving but costly in terms of sensitivity. It may be useful for very pragmatic reviews where there are large numbers of records and speed is important. Alternatively, it may be useful in scoping reviews, where reviewers wish to know the volume or type of research in a particular area, without the need to identify all relevant studies. However, this approach is unlikely to be adequate for reviews requiring high levels of sensitivity.

A more common approach, used for evaluations in the literature [13, 18, 20, 23, 25, 26, 27], and recommended in recent guidance and reviews [30, 31], is to use continued reviewer screening, tool training and reranking until all, or the majority, of included studies are likely to have been found (the “continuous machine training and ranking” approach in Table 1). This is an iterative process, where reviewers screen segments of the total record number and the tool updates predictions and reranks records each time. This approach results in lower workload savings but, in theory, it can achieve maximum sensitivity, making it suitable for use in systematic reviews too.

When using a tool in this way, reviewers must be assured that they have not excluded any relevant studies. To accomplish this when screening with a machine‐learning tool, guidance recommends determining a stop‐criteria, where a decision is made to stop screening, based on the presumption that the majority of relevant records have been included [30]. However, since each review is different in respect to its screening burden, complexity of research question(s), and the nature and volume of included studies, it may be difficult to correctly estimate where this point may be.

In retrospective tool evaluations, where this stop‐criteria has been established, authors have found cut‐points ranging between screening 8% and 93% of records to obtain most included studies [16, 18, 26, 27], with large variation across reviews, even where the same tool is being used. Since the size and complexity of a review has a large impact on the ability of tools to properly rank records [27], this criterion will vary from review to review and, when used prospectively, it may be impossible to set stop criteria at a suitable level.

One screening tool appears to have developed the capability to predict stop criteria by examining the number of excluded records in the spans between obtaining included studies [27]. In this way, the program estimates the likely number of included studies remaining in the unscreened records. In their evaluation, the program performed well, and, in almost every case when a 95% stop‐criteria was applied, more than 95% of included studies were identified. However, this may remain an important barrier in the use of many tools.

### 2.4 Is further research needed?

None of the evaluations of screening tools considered here took account of any additional sources of evidence identification, such as reference checking or citation tracking. Particularly in pragmatic reviews, these methods can be an important source of included studies, making up in part for studies potentially missed through database searches. Where additional searching methods would be part of the desired workflow, it is important to evaluate the accuracy of tools used alongside these methods, as this will better reflect tool accuracy in practice.

However, it is important that evaluations include outcomes that consider the overall impact of tools and not just diagnostic accuracy. These include the impact on the overall review findings and recommendations, and the overall time and cost associated with tool use versus human screening. This may be particularly important where tools are being used to increase efficiency, for example, in rapid reviews, and it is anticipated that some studies will inevitably be missed. Evaluations should incorporate assessment of the difference in findings and conclusions that would be drawn between a review conducted with tool compared to a review conducted using human screening. They should also evaluate the overall time taken for reviews with and without tools and document the time taken for groups to incorporate tools within their workflow. Where this type of evidence shows benefits of tools in terms of time and costs and noninferiority to current practice in other outcomes, this may encourage more widespread tool adoption by reviewers and acceptance by commissioners.

### 2.5 Which one is best?

Despite the large number of available tools and evaluations of their accuracy, little has been published to bring together these evaluations and nothing to compare the accuracy of tools. An extensive systematic review [31] synthesized tool accuracy across studies but made no attempt to compare the accuracy of different tools. This type of comparative assessment is currently limited by the heterogeneity of methods used in different studies.

The literature is full of studies that would be considered noncomparative [13, 14, 15, 16, 17, 18, 19, 20, 21, 22, 23, 24, 25, 26, 27], where, although measures of accuracy are estimated by comparison with a reference standard, there is no comparison with other tools. It is recognized that noncomparative studies are suboptimal for the assessment of relative test accuracy due to differences in patient groups and methods between studies [32]. Because populations and study methods impact test accuracy, the accuracy of different tests cannot be compared because of the heterogeneity across studies. Studies of automated screening tools are an example of this issue, where tool accuracy varies considerably depending on the methods used for tool training and assessment, and the size and complexity of the review test set.

If screening tools are to be compared, comparative studies are needed, where tools are evaluated with the same methods and review test sets. Only two comparative studies were identified [33, 34]. These potentially allow some conclusions to be drawn about the relative accuracy of the included tools, but, even in one of these studies [33], there were variations in tool assessment methods. Additionally, because these evaluations only include a small number of tools, and there is only one publication for each comparison, there is still great uncertainty around the relative accuracy of the range of available tools.

## 3 CONCLUSIONS

Machine learning tools hold considerable potential for accelerating screening in evidence reviews, even when demanding high levels of sensitivity. This technology may lead to significant workload savings, particularly for rapid evidence reviews. Future tool evaluations should include not only measures of accuracy, but the effect of using tools on overall review findings and conclusions, and the time and cost associated with tool integration versus workload saved. Due to the lack of comparative studies in the literature, it is not possible to identify the most accurate tools and more comparative studies may be needed, where tools are compared under the same conditions.

## AUTHOR CONTRIBUTIONS

**Mary Chappell**: Conceptualization; investigation; methodology; writing—original draft. **Mary Edwards**: Conceptualization; methodology; writing—review and editing. **Deborah Watkins**: Conceptualization; writing—review and editing. **Christopher Marshall**: Supervision; validation; writing—review and editing. **Sara Graziadio**: Conceptualization; methodology; supervision; writing—review and editing.

## CONFLICT OF INTEREST STATEMENT

The authors declare no conflict of interest.

## PEER REVIEW

The peer review history for this article is available at https://www.webofscience.com/api/gateway/wos/peer-review/10.1002/cesm.12021.

## Data Availability

Data sharing is not applicable to this article as no datasets were generated or analyzed during the current study.
